# An implementation science approach for developing and implementing a dietitian-led model of care for gestational diabetes: a pre-post study

**DOI:** 10.1186/s12884-020-03352-6

**Published:** 2020-11-03

**Authors:** Nina MELONCELLI, Adrian BARNETT, Susan de JERSEY

**Affiliations:** 1 School of Exercise and Nutrition Sciences, Queensland University of Technology, Kelvin Grove, Queensland Australia; 2 Nutrition and Dietetics, Allied Health, Sunshine Coast University Hospital, Birtinya, Queensland Australia; 3 School of Public Health and Social Work, Queensland University of Technology, Brisbane, Australia; 4 Department of Nutrition and Dietetics, Royal Brisbane and Women’s Hospital, Herston, Australia

**Keywords:** Gestational diabetes, Model of care, I-PARIHS, Implementation science framework, Dietitian

## Abstract

**Background:**

There is strong evidence that women with gestational diabetes mellitus (GDM) who receive a minimum of three appointments with a dietitian may require medication less often. The aim of this study was to evaluate the impact of a dietitian-led model of care on clinical outcomes and to understand the utility of the *integrated* Promoting Action on Research Implementation in Health Services (*i*-PARIHS) framework as a prospective tool for implementation.

**Methods:**

This was a pre-post intervention study measuring outcomes before-and-after changing a gestational diabetes (GDM) model of care and included women with GDM managed at a large, regional hospital in Queensland, Australia. The *i*-PARIHS framework was used to develop, implement and evaluate a dietitian-led model of care which increased dietetic input for women with GDM to a minimum of one initial education and two review appointments. The outcomes were adherence to the schedule of appointments, clinician perspective of the implementation process, pharmacotherapy use, gestational age at commencement of pharmacotherapy and birth weight. Pre- and post- comparisons of outcomes were made using t-tests and chi-squared tests.

**Results:**

Adherence to the dietetic schedule of appointments was significantly increased from 29 to 82% (*p* < 0.001) but pharmacotherapy use also increased by 10% (*p =* 0.10). There were significantly more women in the post-intervention group who were diagnosed with GDM prior to 24 weeks gestation, a strong independent predictor of pharmacotherapy use. Infant birthweight remained unchanged. The *i*-PARIHS framework was used as a diagnostic tool and checklist in the model of care development phase; a facilitation tool during the implementation phase; and during the evaluation phase was used as a reflection tool to identify how the *i-*PARIHS constructs and their interactions that may have impacted on clinical outcomes.

**Conclusions:**

The *i*-PARIHS framework was found to be useful in the development, implementation and evaluation of a dietitian-led model of care which saw almost 90% of women with GDM meet the minimum schedule of dietetic appointments.

**Supplementary Information:**

The online version contains supplementary material available at 10.1186/s12884-020-03352-6.

Contributions to the literature

- Few studies have published specific details on models of care used in gestational diabetes management and even less studies have reported how these models of care were implemented into routine care.
- We developed a novel, dietitian-led model of care for gestational diabetes management that aimed to achieve a minimum schedule of dietetic appointments.
- Our research uniquely documents the prospective use of the *i*-PARIHS framework for changing practice based on clinical guideline recommendations and reports on the utility of the framework in developing, implementing and evaluating a change in gestational diabetes model of care.

## Background

Gestational diabetes mellitus (GDM) is one of the most common disorders in pregnancy and now affects around 12% of pregnancies in Australia [1, 2]. The short-term risks of GDM which includes large-for-gestational age babies, interventional delivery and birth trauma are reduced when GDM is well managed [3, 4]. In the long-term, women with GDM and their infants are at an increased risk of cardio-metabolic disorders and type 2 diabetes mellitus [5]. The primary intervention for GDM is lifestyle changes, including medical nutrition therapy provided by a qualified dietitian [6].

There is strong evidence that women with GDM who are provided with individualised medical nutrition therapy from a qualified dietitian over a minimum of three appointments are less likely to require medication [7–9]. This minimum schedule of dietetic appointments was first recommended in the Academy of Nutrition and Dietetics Nutrition Practice Guidelines [10] and has since been incorporated into the 2015 Queensland Clinical Guideline for GDM [11]. Specifically, the minimum schedule of appointments consists of a 1 h initial counselling session, a minimum of two review appointments and one postnatal follow up visit [11]. Reviews should be scheduled on a two to four weekly basis according to clinical need and further reviews are recommended if pharmacological treatment is initiated [11]. A recent study has demonstrated that the Queensland Clinical Guideline for GDM has been poorly implemented across Queensland Health with fewer than one-third of the hospitals achieving the minimum schedule of dietetic appointments [12].

The translation of clinical guidelines into practice rarely occurs in the short-term without a systematic approach to implementation [13]. Implementation science attempts to address the gap between best-available evidence and clinical practice, and successful implementation involves a multi-faceted approach that considers individual, local and system-based influences on change [14, 15]. As implementation science has grown in popularity, so too have the theories, models and frameworks used to describe, understand or evaluate an implementation process [16].

One well known framework for implementation is the Promoting Action on Research Implementation in Health Services (PARIHS) [17]. The PARIHS framework has undergone considerable content and construct validity, refinement and testing since its original inception in 1998, and has recently been updated to increase its usability as the *integrated* or *i*-PARIHS framework [18–21]. The *i*-PARIHS framework proposes that successful implementation consists of the following three core constructs: the innovation; the context in which the change is to be implemented; and the intended recipients [19]. Each construct is considered at multiple levels (local, organisational and outer layers) and the final element, facilitation, is the ‘active ingredient’ that combines all constructs to enable implementation [19]. The *i*- PARIHS framework is described as a determinant framework used to understand which constructs or domains acts as barriers and enablers to influence implementation outcomes [16]. However, despite the authors’ intention for *i*-PARIHS to be used as a prospective framework to guide implementation processes, it has been predominately used as an evaluation tool [19]. The *i*-PARIHS framework was selected as a development, implementation and evaluation tool for this project in conjunction with the *i*-PARIHS Facilitation Guide due to its alignment with health service research and the specific training undertaken by two of the authors [18].

An opportunity existed to change service delivery to improve women’s access to dietetic input for GDM through the implementation of the minimum schedule of dietetic appointments as recommended by the Queensland Clinical Guideline at a regional hospital in Queensland, Australia [11]. The recommended schedule of care had been successfully implemented using a theory-driven implementation science approach at three other hospitals in Queensland, Australia [8, 9]. The research team for this study saw an opportunity to prospectively use the *i*-PARIHS framework to guide the implementation and so increase the chances of a successful change while uniquely contributing to the existing *i*-PARIHS implementation science literature. The aim of this study was two-fold: to evaluate the impact of the dietitian-led model of care for women with GDM on clinical outcomes; and to understand the utility of the *i*-PARIHS framework to prospectively guide an implementation process.

## Methods

### Study design and participants

This study used a pre-post intervention study design to assess the outcomes of changing a GDM model of care. The *i*-PARIHS theoretical framework was used in the development, implementation and evaluation of the GDM model of care. The study was a collaborative effort between the multidisciplinary team of physicians, dietitians, diabetes educators, administrative staff and the researchers to develop a new ‘innovation’ of a dietitian-led model of care for GDM management. The main goal of the model of care was that women with GDM received the minimum schedule of dietetic appointments. Implementation was considered successful if the model of care was adopted into routine practice and greater than 75% of women achieved the minimum schedule of dietetic appointments.

The study was conducted in a 560 bed, regional Queensland hospital where women with a GDM diagnosis (Table 1) were referred to a specialist diabetes team made up of dietitians, diabetes educators and obstetric physicians for GDM management. This team worked separately to their usual antenatal care team and pharmacotherapy decisions were managed by the diabetes team obstetric physicians. Included women were those with GDM who were diagnosed and referred to the diabetes team during the pre-intervention (30 January to 30 July, 2017) and post-intervention (30 January to 30 July, 2018) periods. To ensure complete data availability, only women who completed their pregnancy journey by birthing at the public hospital were included. The only other exclusion criteria was if the woman required an English-language translator.

**Table 1:** Diagnostic criteria and blood glucose level targets once diagnosed for GDM [11]

**Screening and diagnostic criteria for GDM**	
For women with 2 or more risk factors, [11] offer a 2-h 75 g OGTT in the first trimester. Women without risk factors or with a negative early OGTT, offer a 2-h 75 g OGTT at 24 to 26 weeks.	
OGTT (preferred test for diagnosis)	
One or more of: Fasting ≥92 mg/dL (5.1 mmol/L); 1 h ≥ 180 mg/dL (10 mmol/L); 2 h ≥ 153 mg/dL (8.5 mmol/L)	
HbA1c (if OGTT not suitable)	
First trimester only: ≥ 41 mmol/mol (or 5.9%)	
**Blood glucose level (BGL) targets for GDM patients once diagnosed**	
Self-measured capillary BGLs four times daily (fasting and 2 h post-main meals):	
Fasting ≤90 mg/dL (5.0 mmol/L) plus 1 h after commencement of meal ≤133 mg/dL (7.4 mmol/L) OR 2 h after commencement of meal (preferred) ≤ 121 mg/dL (6.7 mmol/L)	

*GDM* Gestational diabetes mellitus; *OGTT* Oral glucose tolerance test, *HbA1c* Glycated haemoglobin

### The i-PARIHS framework

The *i*-PARIHS framework was used in all phases of the project. As described in the introduction, the core constructs of the *i*-PARIHS framework include: the innovation; the context; recipients and the ‘active ingredient’ of facilitation [19]. The facilitator must have a clear understanding of all constructs, be responsive to characteristics of the innovation and recipients, as well as the potential barriers and enablers to implementation [19].

### Development phase

This six-month phase was used to design the innovation (model of care), collaborate with the recipients (health care team), and understand the influence of the inner and outer contexts. The specific steps taken during the development phase were:

1. A literature review to understand other GDM models of care and specific published barriers and enablers to translating GDM guidelines into practice [22].
2. National and state-wide surveys (published elsewhere [12, 23]) to understand the current practices in GDM management and specific barriers and enablers to implementing the Queensland Clinical Guideline for GDM.
3. Identification of key stakeholders, working party, opinion leaders and champions within the diabetes team to assist with the development and implementation of the model of care.
4. ‘Diagnosis’ and understanding of the i-PARIHS constructs of the innovation, recipients and inner and outer contexts using a checklist adapted from the Facilitation Guide (Supplement 1) in conjunction with the working party. The checklist was used during the development phase to ensure all constructs were fully considered and to diagnose barriers and enablers.
5. Regular meetings with the working party (fortnightly to monthly) to discuss the model of care, patient flow, appointment scheduling and resources required. The meetings additionally allowed clinicians to discuss their concerns and identify potential issues which were solved in a collaborative manner. Adjustments to the model of care were made where necessary.
6. Negotiation for increased dietetic staffing including an additional clinic day dedicated to GDM women.
7. Development of an information sheet for women explaining the team member roles and review schedule for GDM care and who to contact if blood glucose levels (BGLs) were elevated, dependent on whether their GDM was diet-controlled or diet- and pharmacotherapy-controlled.
8. An escalation-of-care flow chart was developed as a decision-making tool for the dietitians to refer women to the physician. Flow-charts outlining the low-and-high-risk models of care were also developed for team members to refer to.
9. Appointment scheduling template and updated instructions for administrative staff.

#### The innovation: a dietitian-led model of care

The innovation for this project was changing the model of care to meet the minimum schedule of dietetic appointments as recommended in the Queensland Clinical Guideline for GDM. It was recognised that evidence is rarely translated in its original form or directly applied from clinical guidelines [19]. Thus, the model of care ‘innovation’ was also developed with consideration of team members’ underlying knowledge and experience and its degree of fit within existing practice and values [19].

Prior to the model of care changing, women with GDM attended a two-hour group education session with the diabetes team dietitian (1 h) and diabetes educator (1 h), within a week of being referred. Both the dietitian and diabetes educator are considered integral to GDM education and management. The dietitian is responsible for the medical nutrition therapy education, counselling and diet individualisation, whereas the diabetes educator is responsible for BGL monitoring education and where necessary, pharmacotherapy education and titration assistance. Both the dietitian and diabetes educator monitor BGL records with the view to escalate care to the team physician where BGLs are above target.

Following the education session, women were asked to self-monitor their BGLs four times a day (fasting and 2 h after each main meal) and record their food intake in a seven-day food diary. One week later, separate diabetes educator and dietitian 30 to 45-min review appointments were conducted. Women with BGLs that exceeded the targets recommended in the Queensland Clinical Guideline (Table 1) two or more times in the same testing period (i.e. fasting or 2 h after the same meal) over a one-week period, were referred to the diabetes team physician who prescribed pharmacotherapy. The diabetes educator would monitor women’s BGLs via phone calls and assisted with medication management as required. Rarely, women would receive a second dietetic review (30 min), primarily for weight management. Women who were able to manage their GDM through diet, were discharged from the diabetes team after a second diabetes educator review and monitored by their usual antenatal team.

Although the main goal of the new model of care was to ensure women received a minimum of three appointments with the dietitian per the Queensland Clinical Guideline, it was during the development phase that team members identified that the 1 week review appointment with both the dietitian and diabetes educator in separate appointments involved task duplication. Specifically, both the dietitian and diabetes educator would check the woman’s BGL record book, identifying whether BGLs were within target. It was decided that the dietitian would become solely responsible for checking BGL records during the one-week review to reduce appointment burden on women. At the end of the development phase, the ‘innovation’ was a dietitian-led model of care where women with GDM would be managed by the dietitian, unless they required pharmacotherapy. Diet-controlled GDM was considered ‘low-risk’ while women requiring pharmacotherapy were considered ‘high-risk’. There were no changes to the way medical nutrition therapy education or counselling was provided with the new model of care.

The differences with the dietitian-led model of care were:

- During the first review following group education, women with GDM were reviewed by the dietitian only during a 45-min face-to-face review. The dietitian individualised their dietary intake based on their food and BGL record. Women were referred to the diabetes team physician when BGLs exceeded the targets recommended in the Queensland Clinical Guideline (Table 1).
- If the physician prescribed pharmacotherapy, the diabetes educator would provide pharmacotherapy education and assistance with self-titration.
- Both low- and high-risk women received a second dietetic review at 34 to 36 weeks gestation. Low-risk women were monitored by the dietitian until the end of their pregnancy via fortnightly email contact where the dietitian would contact the woman by phone if the BGLs exceeded the recommended targets and refer to the team physician. High-risk women were monitored by the physician and diabetes educator via phone or email at intervals based on clinical need.
- The minimum schedule of dietetic appointments for all women were: Two-hour group education (1 h diabetes educator, 1 h dietitian) within 1 week of diagnosis; 45 min face-to-face review (1 week post-group education); and 30 min phone or face-to-face review at 34 to 36 weeks. Additional dietetic appointments could be scheduled as clinically indicated.

#### The recipients

The recipients were the diabetes team members, considered at the individual and collective team level. During the development phase, it was individual recipients (opinion leaders) who identified that many of the tasks undertaken by the dietitian during the first review appointment were replicated by the diabetes educator. This introduced an opportunity to streamline the model of care based on resources, skills and knowledge in the team, and the innovation evolved into a dietitian-led model of care.

#### The inner and outer contexts

There was support within the local and organisational context levels for changing the model of care, so this construct was met with few barriers. Nonetheless, the context barriers and enablers were mapped during the development phase to proactively address potential issues. It was identified that formal and informal leadership support were key enablers to change while historical team cultural issues needed to be overcome. There were no specific characteristics from an external health system context that needed to be considered. As the Queensland Clinical Guideline for GDM had been published state-wide, this was considered as a mandate for the implementation of guideline recommendations.

#### Facilitation

The lead author was the main facilitator and was assisted by an external, experienced facilitator (third author). Both facilitators had undergone specific training in the *i*-PARIHS framework and the Facilitation Guide [18] produced by the *i*-PARIHS authors was a key resource used throughout the project.

### Implementation phase

Education sessions were held in the 3 months prior to commencing the new model of care with the entire diabetes team. The education sessions provided the evidence and context for the proposed changes, as well as updates on specific workflow and referral pathways for the new model. The checklist from the development phase (Supplement 1) was used to guide meeting and education topics and as a reflection tool to better understand where team members’ questions, concerns and discussions sat within each *i*-PARIHS construct. Newsletters and updates via email were distributed bi-monthly to all staff involved prior to changing the model of care, during and after the post-implementation six-month study period.

### Evaluation phase

#### Outcome measures

The outcomes of interest were: adherence to dietetic schedule of appointments (minimum of initial education session and two review appointments); pharmacotherapy use (insulin, metformin or both); gestational age at commencement of pharmacotherapy; birth weight (continuous and categorical variables); and clinician perspective of the implementation process (NoMAD survey instrument [24, 25]). At the beginning of the project, patient satisfaction (survey [26]) and change to diet quality (food frequency questionnaire [27]) were also pre-specified outcomes of interest. However, individuals within the diabetes team did not support the implementation of the patient satisfaction survey due to workload pressures. The food frequency questionnaire was collected from less than 50% of patients and hence it was determined not to proceed with data analysis. The *i*-PARIHS framework was once again used during the evaluation phase as a reflective tool for each of the *i*-PARIHS constructs.

Data were extracted from hospital records for the outcomes of: pharmacotherapy use; adherence to dietetic schedule of appointments; gestational age at commencement of pharmacotherapy; and birth weight. Birth weight for each infant was also categorised according to large-for-gestational (LGA)(> 90th percentile, Australian population) [28] and small-for-gestational (SGA)(< 10th percentile, Australian population) [28] weight. Clinician perspective of the implementation process was measured using the validated 23-item NoMAD survey [24, 25] at the beginning and end of the post-intervention period.

### Statistical analysis

Categorical variables were summarised as frequencies and percents, and continuous variables using mean (standard deviation). The Chi-squared test was used to compare the difference in proportions in the pre-post interventions groups for categorical maternal characteristics, pharmacotherapy use, adherence to dietetics schedule of appointments, and LGA/SGA. The independent samples t-test was used to compare the difference in means before and after changes to the model of care. A post-hoc analysis comparison of pharmacotherapy use and appointments was performed for women diagnosed from 24 weeks after initial data analysis revealed that significantly more women were diagnosed with GDM prior to 24 weeks in the post-intervention group.

Logistic regression was used to calculate adjusted odds ratios and 95% confidence intervals for the risk of requiring pharmacotherapy and delivering an LGA or SGA infant, after changing the model of care. We pre-specified all confounders using a theoretical approach [29] and did not add or remove variables after seeing the results. Statistical analyses were carried out using IBM SPSS version 20.We report our results using the STROBE statement for observational studies [30].

## Results

There were 169 women who were referred for GDM management in the pre-intervention group and 141 women in the post-intervention group. After excluding women who did not birth at the public hospital, 125 women were included in the pre-intervention group and 119 in the post-intervention group. Prior to the model of care changing, dietetic staff resourcing dedicated to GDM was 0.25 full time equivalents (FTE), which was increased to 0.4 FTE after the model of care was implemented. The increase was possible due to increased dietetic staffing allocations which had occurred just prior to the study commencing. GDM staff resourcing for diabetes educators and obstetric physicians remained unchanged. However, in practice, the requirement for diabetes educator resourcing reduced by approximately 0.2 FTE.

### Maternal characteristics

A comparison of the maternal characteristics for the pre- and post-intervention group are reported in Table 2. Of the characteristics which are known predictors for pharmacotherapy use in GDM [31], age and pre-pregnancy body mass index (BMI) were similar between the two groups (Table 2). However, nearly three times more women were diagnosed early (before 24 weeks gestation) in the post-intervention group (21% vs 7.3%, *p* < 0.01), whereas women in the pre-intervention group were more likely to be diagnosed on a fasting BGL (*P* = 0.09), and were also somewhat more likely to have reported a family history of diabetes (*P* = 0.17) (Table 2).

**Table 2:** Maternal characteristics for the pre-and-post intervention groups, before and after the GDM model of care

Maternal Characteristics	Pre-Intervention	Post-Intervention	*P*-Value
Total participants	125	119	
Age (SD), years	32.2 (5.7)	32.7 (5.9)	0.81
Gestational age at diagnosis (SD), weeks	26.9 (3.6)	25.6 (5.6)	< 0.01
Early diagnosis (under 24 weeks), *n* (%)	9 (7.3%)	25 (21.0%)	< 0.01
Diagnosis based on fasting result, *n* (%)	53 (43%)	39 (33%)	0.09
Diagnosis based on 2 or more results, *n* (%)	38 (31%)	27 (24%)	0.20
Parity (SD)	1.1 (1.2)	0.8 (1.1)	0.78
Nulliparous, *n* (%)	50 (40%)	56 (47%)	0.29
PP BMI (SD), kg/m^2^	27.1 (6.4)	27.9 (7.7)	0.19
PP BMI underweight, *n* (%)	6 (5.0%)	3 (2.9%)	
PP BMI normal weight, *n* (%)	47 (39%)	40 (38%)	
PP Overweight, *n* (%)	29 (24%)	29 (28%)	
PP Obese (BMI **≥**30 kg/m^2^), *n* (%)	39 (32%)	32 (31%)	0.81
Indigenous Status, *n* (%)	2 (1.6%)	5 (4.2%)	0.23
Previous GDM, *n* (%)	19 (16%)	24 (20%)	0.37
Family History Diabetes, *n* (%)	58 (49%)	48 (40%)	0.17
Smoking, *n* (%)	9 (8.9%)	15 (13%)	0.37
Pre-pregnancy hypertension, *n* (%)	9 (7.5%)	4 (3.3%)	0.16
Polycystic Ovarian Syndrome, *n* (%)	8 (6.7%)	9 (7.5%)	0.77

*SD* Standard deviation; *PP BMI* Pre-pregnancy Body Mass Index; *GWG* Gestational weight gain; *GDM* Gestational diabetes mellitus

### Adherence to dietetic schedule of appointments

There was a large increase in dietetic appointments in the post-intervention group and the number of women achieving the minimum number of dietetic appointments greatly increased (82% vs 29%, *p* < 0.001). There was also an increase in the mean number of appointments with the diabetes team obstetric physician in the post-intervention group, but a decrease in the number of diabetes educator appointments (Table 3). This trend was similar when only women diagnosed from 24 weeks was analysed (Table 3).

**Table 3:** Dietetic, diabetes educator and obstetric physician appointments, before and after changing a GDM model of care

Appointments	Total			Diagnosis from 24 weeks		
	Pre-Intervention (*n* = 125)	Post-Intervention (*n* = 119)	*P*	Pre-Intervention (*n* = 115)	Post-Intervention (*n* = 94)	*P*
Dietitian, number of appointments, mean (SD)	2.4 (0.8)	3.8 (1.7)	< 0.001	2.4 (0.8)	3.7 (1.4)	< 0.001
Adherence to dietetic schedule of appointments, *n* (%)	36 (29%)	98 (82%)	< 0.001	33 (29%)	79 (84%)	< 0.001
Obstetric Physician, number of appointments, mean (SD)	1.9 (2.5)	2.5 (2.8)	0.08	1.8 (2.3)	2.3 (2.4)	0.11
Diabetes Educator, number of appointments, mean (SD)	3.1 (2.0)	2.6 (2.6)	0.10	3.0 (1.4)	2.3 (2.0)	0.007
Total appointments, mean (SD)	7.4 (4.3)	8.9 (5.1)	0.01	7.1 (3.6)	8.3 (3.9)	0.02

*GDM* Gestational diabetes mellitus; *SD* Standard deviation

### Pharmacotherapy use

Pharmacotherapy use increased in the post-intervention group by 10% (47% v 37%, *P* = 0.10), a clinically significant increase (Table 4). Insulin use more than doubled in the post-intervention group and metformin use was halved (Table 4). The gestational age for commencing pharmacotherapy was also earlier in the post-intervention group (27.8 weeks vs 30.1 weeks, *p* = 0.05) (Table 4). Total pharmacotherapy use in women diagnosed from 24 weeks was 36% vs 44% (*p* = 0.18), an increase of 8% in the post-intervention group (not shown).

**Table 4:** Pharmacotherapy use and maternal and infant outcomes for the pre-and-post intervention groups

Maternal and infant outcomes	Pre-Intervention (*n* = 125)	Post-Intervention (*n* = 120)	Unadjusted *P-*Value	Adjusted *P-*Value*	OR (CI)**
Binary outcomes					
Requiring any pharmacotherapy, *n* (%)	46 (37%)	56 (47%)	0.10	0.15	1.53 (0.86–2.81)
Metformin	20 (16%)	10 (8.3%)			
Insulin	14 (11%)	33 (28%)			
Metformin + insulin	12 (10%)	14 (12%)			
Large-for-gestational age, *n* (%)	10 (8.0%)	12 (10.1%)	0.57	0.56	1.30 (0.54–3.15)
Small-for-gestational age, *n* (%)	10 (8.0%)	12 (10.1%)	0.57	0.81	1.12 (0.45–2.79)
Continuous outcomes					
Gestational age for pharmacotherapy, mean (SD), weeks	30.1 (4.7)	27.8 (6.8)	0.05		
Infant birthweight, mean (SD), grams	3352 (499)	3290 (470)	0.32		

*Confounders in logistic regression modelling: Requiring any pharmacotherapy (maternal age > 30 years, pre-pregnancy BMI > 30 kg/m^2^, previous GDM, diagnostic fasting > 5.2, early diagnosis < 24 weeks gestation, family history of type 2 diabetes mellitus); large-for-gestational age (pre-pregnancy BMI > 30 kg/m^2^); small -for-gestational age (smoking, during pregnancy)

** OR, 95% confidence intervals from logistic regression models using the pre-intervention as the reference group

### Infant birthweight

The mean infant birthweight decreased by 62 g in the post-intervention group (Table 4). Large-for-gestational age and SGA infants increased by 2% in the post-intervention group (Table 4). Interestingly, of the ten LGA infants in the pre-intervention group, 40% (*n* = 4) of the mothers were treated with pharmacotherapy whereas in the post-intervention group, 67% (*n* = 8) of the mothers were treated with pharmacotherapy.

### NoMAD survey instrument

Five of the 11 staff involved in the GDM team completed the NoMAD survey both before and after the change, a response rate of 45%. Due to the anonymity of the survey, it is not known whether they were the same five staff each time. There was no change in the responses from before and after the model of care was implemented for 59% (*n* = 13) of the questions. Specifically, all responses were positive before and after the model was changed, indicating that the staff felt the model of care was familiar and they had an understanding of the purpose of the model of care, how it affected the nature of their work and the potential value of the model. There was also agreement that the staff felt there were key people driving the model of care forward, that participating in the model was a legitimate part of their work and they would continue to support the model.

Prior to the model of care changing, one of the five staff did not agree with the following statements: they could easily integrate the model of care into their existing workload; sufficient training was provided to enable staff to implement the model; and that staff agree the model of care is worthwhile. Two staff did not agree that sufficient resources were available to support the new model of care. After the model had been implemented one respondent did not agree that sufficient resources were available, and a separate respondent did not agree that they could modify how they worked with the model of care.

### The i-PARIHS framework

The study processes as they occurred within the *i*-PARIHS framework is summarised in Table 5, including a description of the pre-post models of care, literature review findings and facilitation activities. It was discovered that the *i*-PARIHS framework was most useful during the development phase to understand specific barriers and enablers within each construct and as a diagnostic tool and checklist when developing the innovation and preparing for implementation. While the *i*-PARIHS framework was also useful as a reflection tool in the evaluation phase, particularly within each construct, the authors did not feel that the *i*-PARIHS framework uniquely contributed to evaluation as it did with the development phase.

**Table 5:** The development and implementation of a GDM dietitian-led model of care using the i-PARIHS framework

	Innovation	Recipients	Context	Facilitation activities
Development Phase				
Overview	Starting point: • Minimum schedule of dietetic appointments (Queensland Clinical Guideline for GDM) • Goal to increase women’s access to dietetic support and reduce pharmacotherapy requirements. Organisational fit: • Task duplication identified • Low and high-risk models of care (diet-controlled vs pharmacotherapy + diet) • Models of care: Low risk as dietitian-led, high risk as diabetes educator and physician led • Increased surveillance for low-risk GDM patients (due to third dietetic appointment) • Timing of appointments and changes to ongoing monitoring of all women with GDM. Supporting material: • Escalation of care flow chart for dietitians • Low and High-risk model of care summary flowcharts • Updated patient information • Pre-implementation checklists	Recipients (Staff): • Diabetes team members: Dietitians, Diabetes Educators, Nursing Unit Manager, Clinical Nurse Consultant, Director of Endocrinology, Obstetric Physicians, Administration Officers. • Working party: Clinical Nurse Consultant (opinion leader/ authority), Dietitians (champions/ opinion leader), Nursing Unit Manager (authority), Diabetes Educators (champions)	Local: • Increasing GDM diagnosis requiring efficient model of care • Task duplication within the team • Leadership change Organisational: • Change to organisational structure. • Period of transition (opening of new hospital). External Health Systems: • State-wide publication of Clinical Guideline for GDM (2015)	Problem identification: • Clinical guideline recommendation for MNT not met Acquiring/appraising evidence: • Literature review [7, 8, 22, 32, 33] • Prior research (Surveys) [12, 23] • Service mapping Consensus building: • Stakeholder mapping and engagement • Team meetings • Goal setting • Local context assessment: • Diagnosis using i-PARIHS guidance • Model of care development meetings • Working party contributions
Barriers	• Staff resourcing • Education/knowledge • Managing schedule of appointments	• Some resistance to change (minor) • Competing interdisciplinary priorities • Differences of opinion • Perceived workload pressures • Motivation and engagement	Local: • Historical resistance to change • Team culture Organisational: • Period of high organisational change and transition	Project management: • Increase to dietitian FTE/ clinic days • Appointment template changes • Working party meetings • Newsletters/ email updates Improvement methods: • Professional development sessions • Team meetings Conflict management and resolution: • Leadership involvement • One-on-one meetings Team building • Team meetings • Acknowledging key contributions
Enablers	• Strong evidence-base • State-wide guidelines • Well-established team • Dedicated researcher	• Leadership support • Local opinion leaders/ champions • Minimal disruption to usual workflow • Individuals and team able to implement change • Low staff turnover	Local: • Team autonomy • Leadership support Organisational: • Executive support • Alignment with organisational and research priorities External Health System: • State-wide mandate	Team building: • Acknowledging enablers • Feedback
Implementation Phase				
Intervention/ change in practice	• New schedule of dietetic appointments and reduction of diabetes educator appointments • Dissemination of supporting materials	• Increase to dietetic staffing time for GDM • Procedures and policies to inform local system changes	• Procedures and policies to inform local system changes • Informed stakeholders and executive of change to model of care	Communication and feedback: • Fortnightly meetings • Newsletters/ email updates Conflict management and resolution: • One-on-one meetings • Leadership involvement
Evaluation Phase				
Successes	• Adherence to schedule of dietetic appointments (29% vs 88%)	• NoMAD survey: familiar, understanding of purpose, support for the model of care, change in negative perceptions	Local: • Dietitian-led model of care adopted as standard practice	
Confounders	• Appointment timing deviated from original Academy of Nutrition and Dietetics Nutrition Practice Guidelines • Initial education as group rather than individual • Fidelity: patient satisfaction survey not implemented • Sustainability: FFQ data collection not completed at second review	• Lack of perceived value for understanding patient satisfaction and FFQ • Significant differences in baseline characteristics between pre-and-post intervention groups (early diagnosis, family history of diabetes mellitus, previous diagnosis of GDM)	Local: • Increased surveillance of women with GDM to the end of their pregnancy	Communication and feedback: • Newsletters/ email updates • Post-implementation presentation to team members

*GDM* Gestational diabetes mellitus; *FFQ* Food frequency questionnaire

## Discussion

The aims of this study were to evaluate the impact of a dietitian-led model of care for women with GDM on clinical outcomes and to understand the utility of the *i*-PARIHS framework as a prospective tool in an implementation process. We used a theoretical-approach [19] to develop, implement and evaluate the changes to the GDM model of care in order to create real change in the care of women with GDM. As a result, adherence to the minimum schedule of dietetic appointments (one initial education and at least two review appointments) was greatly increased after implementing the model of care changes.

Despite improving dietetic input, requirements for pharmacotherapy increased in the post-intervention group by 10%, a clinically significant increase. It is thought this result was due to important differences between the two groups rather than the model of care itself. A strong independent predictor of requiring pharmacotherapy to treat GDM is an early diagnosis (before 24 weeks gestation) [31, 34, 35]. The number of women diagnosed early in the post-intervention group was almost three times that in the pre-intervention group. There were also differences in the percentage of women who had previously been diagnosed with GDM, with 4% more women in the post-intervention group reporting a previous GDM diagnosis [31]. However, despite adjusting for these confounders in logistic regression modelling and performing a post-hoc analysis on women diagnosed from 24 weeks, there was still a clinically significant increase in women requiring pharmacotherapy after increasing dietetic input.

One of the biggest factors that likely influenced pharmacotherapy use was increased surveillance by the diabetes team. Prior to the model changing, women who achieved their BGL targets at the first review appointment were often discharged from the diabetes team and it was expected their BGLs would continue to be monitored by their usual antenatal care providers. Once the model of care changed, women in the post-intervention group were followed by the diabetes team to the end of their pregnancy and women with elevated BGLs beyond the first review appointment were more likely to be picked up and referred to the obstetric physician for pharmacotherapy.

There were other factors that may have impacted pharmacotherapy requirements for women in the post-intervention group. The timing of the dietetic appointments was based on consultation with the implementation recipients (diabetes team members) and most women were seen at fixed intervals: initial education within a week of diagnosis; first review appointment a week later; and second review appointment between 34 and 36 weeks gestation. On reflection, appointments with a dietitian are likely to be more effective in the early stages of diagnosis where women are most able to make positive dietary and lifestyle changes with intensive support [6]. While close attention was paid to the adherence to the minimum schedule of dietetic appointments, we were unsuccessful at implementing the timing recommended in the Academy of Nutrition and Dietetics Nutrition Practice Guidelines [10]. By scheduling the second review appointment at 34 weeks gestation, there was effectively little difference between the amount of dietetic support in the pre-and post-intervention groups in the weeks following diagnosis.

Prior research on dietetic input for women with GDM [7], including studies undertaken at three Queensland hospitals [8, 9], has shown that women who achieve a minimum of three dietetic appointments are less likely to require pharmacotherapy. In the Queensland studies, only one of the three hospitals achieved a similar level of adherence to the minimum schedule (one initial and two reviews) as our study, yet all hospitals achieved clinically relevant reductions in pharmacotherapy [8, 9]. Interestingly, one of the hospitals only marginally improved their adherence to the minimum schedule of appointments (4.8% of women received at least 3 appointments vs 3.4%), yet still achieved a 9.1% decrease in pharmacotherapy requirements [9]. However, the number of women receiving individual dietetic appointments as the initial consult increased from 2 to 43%, indicating that individual education may be a more important than the number of appointments women receive [9]. In this study, the initial consult for women was always via group education, therefore it is likely our results may have improved had we been able to change this to an individual format. Furthermore, previous research [7–9] did not evaluate outcomes for women diagnosed before 24 weeks, a predictor for pharmacotherapy requirements, as previously described. Finally, in the study by Reader, Splett and Gunderson assessing the impact of the Academy of Nutrition and Dietetics Nutrition Practice Guidelines, the overall results demonstrated a decrease in insulin use at sites adhering to the Nutrition Practice Guidelines but this difference was not detected at sites where women with GDM were managed by a specialist diabetes team [7]. In the present study, both the pre- and post-intervention groups were managed by a specialist diabetes team. It is likely that the interplay between the local context (specialist diabetes centre), the characteristics of the women, and the initial education session and timing of appointments (innovation) meant we were unable to achieve a reduction in pharmacotherapy as predicted.

Despite the increased pharmacotherapy use following the model of care changes, the overall development and implementation of the model of care was considered successful as defined by achieving adherence to the minimum schedule of dietetic appointments for more than 75% of women. According to the NOMAD survey, the changes were easily understood and valued by the staff who responded and since the completion of this study, the model of care has been accepted as standard practice within this team.

The prospective use of the *i*-PARIHS framework provided structure and guidance during the development and implementation phases of this study while highlighting the important role of the facilitator. During the evaluation phase, it also provided the researchers with key constructs to consider as influencing factors to the clinical outcomes. In contrast to much of the previous research which has focused on the retrospective application of the *i*-PARIHS framework, we found *i*-PARIHS was most useful during the development phase as a diagnostic tool and checklist. However, during the evaluation phase we were able to reflect on the influencing factors to the outcomes within each *i*-PARIHS construct.

Despite using a theoretical approach to implementation, not all aspects of changing the model of care were adhered to, demonstrating some of the difficulties with real world health services research. For example, collection of the food frequency questionnaire was incomplete and the patient satisfaction survey was never implemented, highlighting issues with fidelity, acceptability, and sustainability. It is possible the recipients of the implementation did not understand the value of this data, an aspect overlooked by the researchers. Furthermore, collaboration and negotiation with recipients was an important change management strategy but this negotiation meant specifying the follow-up schedule of appointments, resulting in a deviation from the Academy of Nutrition and Dietetics’ recommendations. Attempting to balance best available evidence with organisational ‘fit’ may have impacted the success of the outcome of pharmacotherapy use.

The main limitation of the study was the small sample size and differences in baseline characteristics between the two groups, thus we were unable to determine the true change in the outcome of pharmacotherapy. Due to its low response rate, the results of the NoMAD survey cannot be considered representative of all staff recipients. We were also limited in our evaluation with the exclusion of the pre-specified outcomes of the patient satisfaction survey and the food frequency questionnaire. It is possible that bias was introduced due to the main facilitator also being responsible for the evaluation and interpretation of data, although most data collection was performed by a research assistant who was not involved in data analysis. Most data were collected from chart entries, which has the potential to introduce inaccuracies due to incorrect data entry. Our study reported on a small group of pregnant women, residing in South Eastern Queensland and cannot be considered generalisable to the wider Queensland or Australian population.

## Conclusion

Translating evidence into practice requires a theory-driven approach to ensure sustainable change. The *i*-PARIHS framework can be used prospectively as both a diagnostic tool and checklist as it highlights specific constructs to consider before implementation should be attempted. For this study, active facilitation and understanding barriers and enablers to change were key to increasing adherence to the minimum schedule of dietetic appointments as recommended by the Queensland Clinical Guideline for GDM. While pharmacotherapy use was not reduced in this instance, using the *i*-PARIHS framework allowed for the understanding of influencing factors that may have otherwise been overlooked had the study been reliant solely on clinical outcomes. Medical nutrition therapy and lifestyle modification are key factors to successfully managing GDM and we propose that a dietitian-led model of care and adherence to the minimum schedule of dietetic appointments is feasible for routine GDM care.

## Supplementary Information

**Additional file 1: Supplement 1.** The i-PARIHS facilitation checklist and reflection tool as adapted from *Implementing evidence-based practice in health care – A facilitation guide* by Gill Harvey and Alison Kitson.

## Data Availability

The datasets generated and/or analysed during the current study are not publicly available due to restrictions imposed through the Public Health Act 2005 waiver of participant consent but may be available from the corresponding author on reasonable request.

## Abbreviations

i-PARIHS: *Integrated* promoting action on research implementation in health services
BGLs: Blood glucose levels
BMI: Body mass index
FTE: Full time equivalents
GDM: Gestational diabetes mellitus
LGA: Large-for-gestational age
SGA: Small-for-gestational age
