# Supplementary material for: An implementation science approach for developing and implementing a dietitian-led model of care for gestational diabetes: a pre-post study
Source: BMC Pregnancy Childbirth. 2020 Nov 3;20:661. doi: 10.1186/s12884-020-03352-6 (PMC7607700; doi:10.1186/s12884-020-03352-6)
Supplement: Supplementary file 1 — Additional file 1: Supplement 1. The i-PARIHS facilitation checklist and reflection tool as adapted from Implementing evidence-based practice in health care – A facilitation guide by Gill Harvey and Alison Kitson. [file 12884_2020_3352_MOESM1_ESM.docx]

**Supplement 1:** The i-PARIHS facilitation checklist and reflection tool as adapted from *Implementing evidence-based practice in health care – A facilitation guide* by Gill Harvey and Alison Kitson

|  | Checklist/ Reflection |
| --- | --- |
| **Characteristics of the innovation** |  |
| Who is likely to be affected by the proposed innovation? |  |
| What is the underlying evidence for the proposed innovation or change? |  |
| Is it derived from research, clinical consensus, patient views, local information/ data or a combination of these |  |
| Is it viewed as rigorous and robust? |  |
| Is there a shared view about the evidence? |  |
| How well does it 'fit' the local setting? |  |
| Is it likely to be accepted or contested by those people who have to implement it? |  |
| Is the evidence packaged in an accessible and usable form (e.g. a clinical guideline, care pathway or algorithm)? |  |
| Will people be able to see easily and clearly what is proposed in terms of clinical practice and the process of patient care? |  |
| How much novelty does the evidence introduce? |  |
| Will it require significant changes in the processes and /or systems of care delivery? |  |
| Will it present and challenge to people’s ways of thinking, mental models and relationships? |  |
| What are the implications of this in terms of the likely boundaries that will be encountered? |  |
| Will a knowledge transfer, translation or transformation strategy be required? |  |
| Does it offer advantage over the current way of doing things, for example: |  |
| Will it enhance patient experience? |  |
| Could it introduce greater efficiency in the provision of care? |  |
| Will it help to remove bottlenecks in the care process? |  |
| Is there potential to test out/ pilot the introduction of the evidence/ innovation on a small scale in the first instance? |  |
| **The recipients** |  |
| ***Motivation and ability to change: Individual level*** |  |
| Do individual members of the team want to apply the change in practice? |  |
| Do they perceive the proposed change as valuable and worthwhile? |  |
| Do they see a need to make the change? |  |
| Is the change consistent with their existing values and beliefs? |  |
| Are there individuals who function as local opinion leaders? Will they be supportive or obstructive in terms of introducing he proposed change? |  |
| Are individual members able to implement the proposed change? |  |
| Do they understand what the change entrails? |  |
| Is it within their current level of knowledge and skills? |  |
| Will additional training and development be needed? |  |
| Do people understand the modifications that will be needed to routine practice and how to change and embed these? |  |
| Do individuals have the necessary authority to carry out the proposed changes? |  |
| Have key individuals whose support is needed been identified? Are they engaged in discussing and planning implementation? |  |
| ***Motivation and ability to change: Team level*** |  |
| At a collective level, does the team want to apply the change in practice? |  |
| It the proposed change seen as valuable and worthwhile? |  |
| Do they see a need to make a change? |  |
| Is there a shared view or are there differences of opinion (e.g. between key individuals or between different professional groups and communities of practice)? |  |
| Is there existing data that can be used to highlight the potential for improvement? Or can you collect data for this purpose? |  |
| Are the team able to implement the proposed change |  |
| Do they understand what the change entails? |  |
| Is it within their current level of knowledge and skills? |  |
| Will additional training and development be needed? |  |
| Does the team understand the modifications that will be needed to routine practice and how to change and embed these? |  |
| Does the team have the necessary authority to carry out the proposed changes? |  |
| Is there good inter-professional collaboration and teamwork - between professional groups and between clinical staff and managers? |  |
| Will support be needed to develop more effective collaboration and teamwork? |  |
| Are the potential barriers to implementation known? Are there strategies in place to address these? |  |
| Are the resources available to support the implementation process, for example: time and/or financial support for new skills development, new equipment, expert support and advice? |  |
| **The inner context** |  |
| ***The local context*** |  |
| Who are the formal and informal leaders at a local level? |  |
| Are they likely to be supportive of the proposed change? |  |
| Are they helping to create a facilitative context through providing motivation and support, creating a vision and reinforcing the change process? |  |
| Is there a distributed and devolved style of management? |  |
| Is there a culture that supports innovation and change? |  |
| Do staff feel actively involved in decisions that affect them? |  |
| Are staff trusted to introduce new ideas into practice? |  |
| What is the past experience of introducing changes at a local level? |  |
| Are there mechanisms in place to support learning and evaluation and to embed changes into routine practice (e.g. regular team meetings, audit and feedback processes, professional development opportunities, and performance review systems?) |  |
| ***The organisational context*** |  |
| Do the evidence/ innovation and the changes proposed align with the strategic priorities for the organisation? |  |
| Has the support of key individuals and leaders within the organisation been sought and secured? |  |
| Is there a culture that supports innovation and change? |  |
| Is there a history of successful and sustained change within the organisation? |  |
| Does the organisation have systems and process in place that support innovation and change (e.g. effective information and communication systems, opportunities for networking and learning across departments/ teams)? |  |
| Do the senior management team actively seek opportunities for improvement and encourage ideas and feedback from patients, the public and staff? |  |
| Are there mechanisms in place for embedding changes in routine practice (e.g. formal policies and procedures)? |  |
| **The outer context** |  |
| Do the evidence/ innovation and the changes proposed align with the strategic priorities for the wider health system (e.g. in terms of current health policy, national priorities for action and improvement)?? |  |
| Are there incentives in the wider health system that reinforce the proposed change (e.g. pay for performance schemes, regulatory requirements, etc.)? |  |
| Are there inter-organisational networks (e.g. specialised clinical networks) that will be helpful in terms of supporting the proposed changes? |  |
| How much stability/ instability is there in the wider health system? |  |
| Is this likely to influence the implementation project? |  |
